# Editorial: Platelets as Immune Cells in Physiology and Immunopathology

**DOI:** 10.3389/fimmu.2015.00274

**Published:** 2015-06-03

**Authors:** Olivier Garraud

**Affiliations:** ^1^EA3064, University of Lyon Saint-Etienne, Saint-Etienne, France; ^2^Institut National de la Transfusion Sanguine (INTS), Paris, France

**Keywords:** platelets, inflammation, infection, transfusion, immunity

Blood platelets are essential for the earliest stages of coagulation, namely, primary hemostasis. They adhere to damaged vessel endothelium, stick to each other (aggregate), and form clots; this prevents bleeding. For most physicians, those attributes of platelets are exactly what they learned in medical school years ago, and this basic knowledge seems quite enough to allow a valid therapeutic strategy when numbers or hemostatic functions of platelets are aberrant. In some cases, this consists in prescribing anti-platelet drugs (aspirin or more sophisticated drugs) to prevent overly active clotting in cardiovascular and metabolic dysfunctions. In other instances, this consists in prescribing platelet transfusions when the platelet count is dangerously low (or – in exceptional occasions – when platelets are dysfunctional). It could be as simple as that, but in fact, it is often not, because platelets are more versatile than initially thought (or expected) and some modification is needed in many cases (1). To cite only one example, anti-viral treatment of HIV infection causes atheroma and platelet deposition, emphasizing the recently recognized inflammatory function of platelets (2, 3); anti-platelet therapy seems a likely approach, but this is not current practice yet.

Thus, let us imagine that a scientific magazine writer decides to contribute a paper emphasizing novel advances in platelet research; the journal’s instructions are: no more than four key points, a concise style, and only issues that can be understood by a large community; and – icing on the cake – a translation into today’s or tomorrow’s therapeutics. What would he/she insist on?

The proposed four points, which in our opinion, are either really new or newly rediscovered (after having been buried for decades and perhaps completely forgotten) would be:

– Not only are platelets genuine cells but also are they intelligent cells, as they can sense dangers differentially (4, 5).
– Despite platelets have been suspected to be inflammatory cells as soon as in the early 70s’, this opinion either has been ignored or faded (6). Platelets indeed participate in innate immunity and they can influence adaptive immunity (7–9); they are “licensed” as highly potent pro-inflammatory cells (10).
– Platelets have a remarkable ability to sense and bind microbial agents, in particular, pathogenic viruses, and foremost bacteria (11, 12); interestingly enough, this property has been recognized in the early 70s’ (13) but not exploited since recently (14).
– Platelets have more than one cell partner (the endothelial cell) as they intimately interact with the leukocyte, not only at different phases of the clot formation but also in tissue pathology (15, 16); this is also an issue, which is rediscovered after having been under-acknowledged according to its importance (17).

Why are all four key points really interesting for the medical community, and – beyond – to the patient community?

Concisely, we suggest three reasons:

– First, these findings lead to revisiting the essential functions of platelets. While platelets were principally considered relevant to vascular pathology (vessel injury and bleeding), today they are also considered as sentinels along the vascular tree, detecting insults and making daily repairs. Importantly, platelets perform an immune function as danger sensors, detecting circulating viruses and bacteria.
– Second, because they are non-nucleated, and mere fragments of the megakaryocyte, platelets were thought to be terminally differentiated cells, limited in function, fully equipped with static content; only one option remained to enrich their functions: to borrow glycoproteins from the environment. Recent evidence is that platelets can give rise to progeny platelets (18). Do these daughter cells possess identical capabilities to those of the mother cell? And are there no distinct subsets of platelets with different functions *in vivo*, as might be indicated by varying capacity for differential cytokine/chemokine secretion? Further, platelets are capable of using RNA to make secreted proteins (19, 20), an issue, which was suspected as in the late 60s’ (21) and then disregarded (it is not fully consensual yet): not so a static dead-end cell after all!
– Lastly, platelets have been considered for some time as sentinels in severe clinical infection and particularly in sepsis (14). Maybe platelets are not just sentinels, but one among the primary targets of infectious pathogens, contributing to severe organ failure, especially because of their intimate relationship with leukocytes (22). Platelets were recently shown to infiltrate joints and cause serious inflammatory damage. Collectively, these observations call for revisiting at least partly the therapy of certain auto-inflammatory and infectious disease: what about anti-platelet drug use? Oh yes, some are very cheap such as aspirin: but is this effective and safe? In all, anti-platelet therapy reveals itself far more complex and nuanced than previously considered (23).

In aggregate, platelets span the classic field of hemostasis and thrombosis and the novel field of immunology and inflammation. Even transfusion medicine gurus are confused. Most were taught that low platelet counts below a given threshold (ranging from 10,000 to 30,000/μL of blood) require a platelet transfusion; now, they are kindly advised that all platelet transfusions are not equal and some transfusions may well be more pro-inflammatory than others (24, 25). How can they choose? They cannot, because the blood bank has made the choice for them: they are left with some confusion and concerns for their patients. Of note, this last paragraph would certainly not have been appropriate for the hypothetical science writer, because it does not speak to current reality of medical practice. Well, maybe not yet; but physicians and scientists are making significant progress in rendering platelet transfusion definitely much safer in terms of reduction of immunological hazards, and detecting genetic predisposition to harm (26, 27). Thus, we have the new general understanding that platelets are not that simple, after all, and that there may well be much more to learn again.

## Conflict of Interest Statement

The author declares that the research was conducted in the absence of any commercial or financial relationships that could be construed as a potential conflict of interest.
